# The assesment of relationship between the angulation of impacted mandibular third molar teeth and the thickness of lingual bone: A prospective clinical study

**DOI:** 10.4317/medoral.22596

**Published:** 2018-12-24

**Authors:** Dilek Menziletoglu, Melek Tassoker, Bozkurt Kubilay-Isik, Alparslan Esen

**Affiliations:** 1 Dr, Necmettin Erbakan University, Faculty of Dentistry, Department of Oral and Maxillofacial Surgery, Konya/Turkey; 2 Dr, Necmettin Erbakan University, Faculty of Dentistry, Department of Oral and Maxillofacial Radiology, Konya/Turkey; 3 Prof Dr, Necmettin Erbakan University, Faculty of Dentistry, Department of Oral and Maxillofacial Surgery, Konya/Turkey; 4 Associate Prof, Necmettin Erbakan University, Faculty of Dentistry, Department of Oral and Maxillofacial Surgery, Konya/Turkey

## Abstract

**Background:**

Our purpose was to investigate the relationship between the angulation of mandibular third molars and the thickness of the lingual bone, which can affect the risk of lingual nerve damage during lower third molars surgical extraction.

**Material and Methods:**

This study consisted of 104 patients (42 males and 62 females), aged between 18-42 years (24.67 ± 6.11 years). Cone Beam Computed Tomography (CBCT) images were taken for preoperative assessment. The teeth were divided into four groups according to their positions: mesioangular, distoangular, vertical and horizontal. Lingual bone thickness around impacted teeth were measured at three points: cementoenamel junction (CEJ) of the mandibular second molar, mid-root of the impacted third molar, and apex of the impacted third molar root. Two predisposing factors of lingual nerve damage were recorded: lingual bone perforated by the impacted tooth and lingual bone thinner than 1 mm. Additionally, buccolingual angulations of the teeth in each group were measured. Impacted mandibular third molars were removed in usual way. One week after surgery, the patients were evaluated regarding lingual nerve paresthesia.

**Results:**

None of the 104 patients experienced paresthesia, including the ones who had teeth with close proximity with lingual nerve. The mean thickness of bone was 1.21±0.63 mm at CEJ of the second molar; 1.25±1.02 mm at the mid-root; and 1.06±1.31 mm at the apex. Horizontally impacted teeth had thinner lingual bone at mid-root level (*p*=0.016). Buccolingual angulated teeth were more often associated with perforated lingual bone (*p*=0.002). Buccolingual and mesial/distal angulation had negative correlation with lingual bone thickness (*p*<0.05).

**Conclusions:**

As the buccolingual and mesiodistal angulations increase, lingual bone thickness decreases. Horizontally impacted teeth seemed to compromise the integrity of the lingual bone more than impacted teeth in other positions. During the surgery, thin or perforated lingual bone may result in displacement of the impacted tooth lingually.

** Key words:**Lingual bone, impacted third molar, cone beam computed tomography, angulation, paresthesia.

## Introduction

During extraction of impacted mandibular third molars, lingual nerve may be damaged. The incidence for this complication has been reported between 0.5% and 2.6% (1). Age of the patient (2), surgeon’s experience (3), trauma to the soft tissue during inferior alveolar nerve block anesthesia (4), impaction pattern of the tooth, retraction of the lingual flap, overall difficulty of the surgery (2,5), amount of the bone removal (6), suturing (7), exposing the lingual nerve during the operation (5) can affect the situation. Most of these nerve damages heal spontaneously. However, some cases advance to permanent paresthesia, hypoesthesia, dysesthesia, speech or chewing disorders that can affect the quality of life (8).

For the preoperative radiologic examination of lower impacted third molars, panoramic films are routinely used. In addition, other imaging techniques have also been suggested. Although they are not routinely used, some authors have recommended magnetic resonance imaging (MRI) (9) or ultrasonography (10) to assess the lingual nerve. High resolution 3-T MRI imaging allows an accurate study of the trigeminal nerve and especially of its branches. The knowledge of the course and of the anatomic relationships of these nerve bundles with surrounding structures, as well as of the anatomical variants, allow oral and maxillofacial surgical planning thus reducing the risk of nerve damage (11). Cone beam computerized tomography (CBCT) can also be used to evaluate the relationship between lower third molars and lingual nerve. CBCT technique cannot demonstrate the lingual nerve itself but if preoperative CBCT images reveal that a cortical bone plate does not exist on the lingual aspect of the lower third molar, the risk for damaging the lingual nerve is higher (12).

In this study, we aimed to investigate the relationship between the angulation of mandibular third molars and the thickness of the lingual bone.

## Material and Methods

-Study design and sample

The study protocol was approved by the local ethics committee (decision no: 2017/04). The study conformed to the guidelines laid out in the Declaration of Helsinki and written consents were obtained from all participants.

This prospective cohort study consisted of 104 patients (42 males and 62 females), aged between 18-42 years (24.67 ± 6.11 years). Patients were followed for post-operative paresthesia existence after impacted third molar surgery. The CBCT scans were acquired using a 3D Accuitomo 170® machine (Morita, Kyoto, Japan) with 10 cm x 10 cm FOV size and operated at 90 kV and 12 mA. The slice thickness was 1 mm and voxel size 0.25 mm3 for this FOV.

Fully erupted third molars, developing third molars with incomplete root formation, dentoalveolar deformities, pathologic conditions in third molar region, patients with missing second or first molars, pericoronal pathology and medical or neurological abnormalities associated with third molars were excluded from the study.

The teeth were divided into four groups related with their position on CBCT images, i.e., mesioangular, distoangular, vertical and horizontal according to Winter (13). The other categories in Winter’s classification were excluded.

-Evaluation of images

Axial, coronal, and sagittal CBCT slices were used to measure the bone thickness around impacted teeth and calculate the third molar angulations in mesiodistal and buccolingual aspects. All CBCT images were evaluated by the same oral and maxillofacial radiologist in a dark room and in the same computer [Intel® Xeon® E5-2620, 2.0 GHz; NVIDIA quadro 2000; 32” Dell T7600 workstation with resolution of 1280 x 1024 pixels, 8 GB memory, Windows 7 operating system] with use of the i-Dixel software Ver. 2.0 (J. Morita MFG. Co.).

Measurements of the lingual cortical bone thickness around impacted teeth were performed at three points according to previously described method of (14).

**Figure 1:**
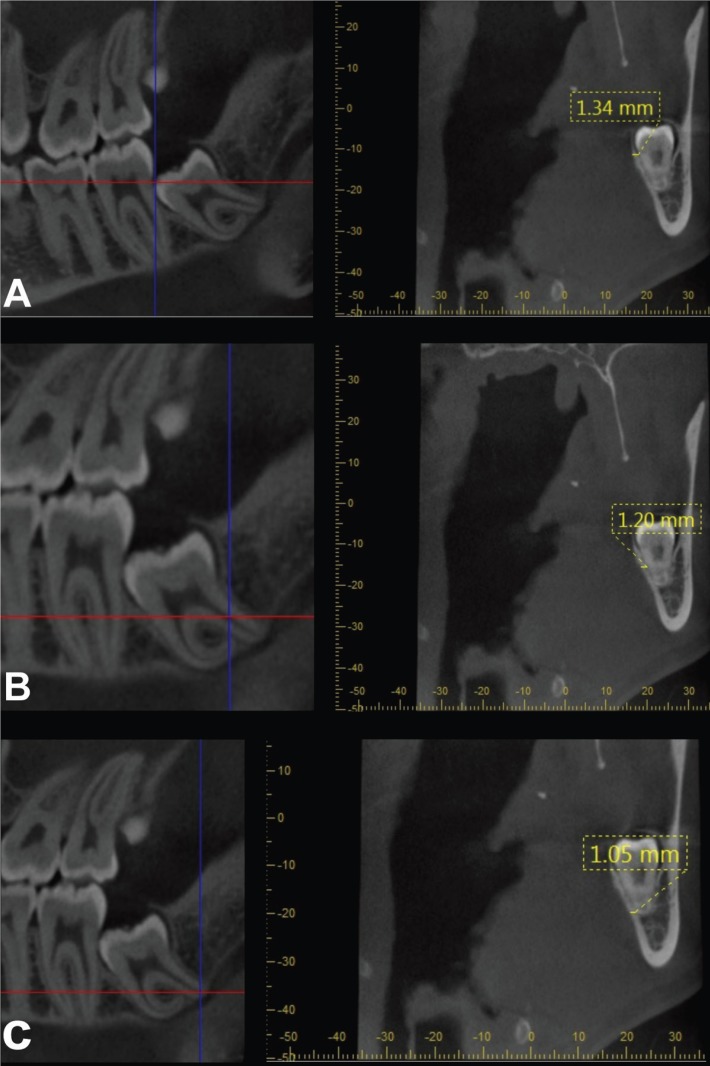
A-Lingual cortical bone thickness at the level of cementoenamel junction of the mandibular second molar. B-Lingual cortical bone thickness at mid-root level of the impacted third molar. C- Lingual cortical bone thickness at the apex of third molar.

1. Cementoenamel junction (CEJ) of the mandibular second molar (Fig. 1A)

2. Mid-root of the impacted third molar (Fig. 1B)

3. Apex of the impacted third molar root (Fig. 1C)

**Figure 2:**
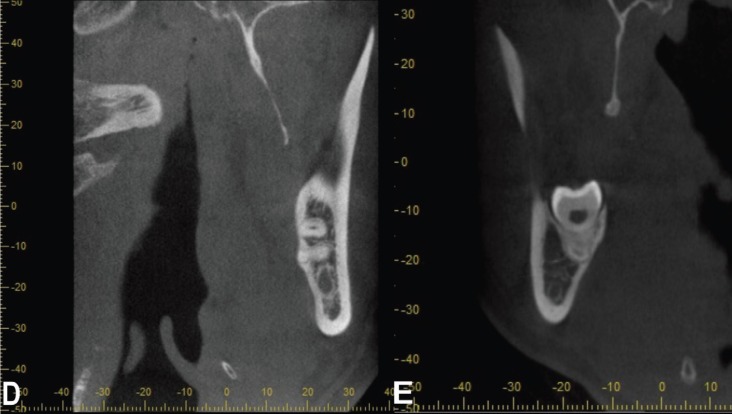
D- Lingual cortical plate perforation. E- Lingual cortical bone thinning (<1mm).

The possible leading factors for lingual nerve damage such as “lingual plate perforation” (Fig. 2D) and “bone thinning that was less than 1 mm thick” (Fig. 2E) were recorded.

**Figure 3:**
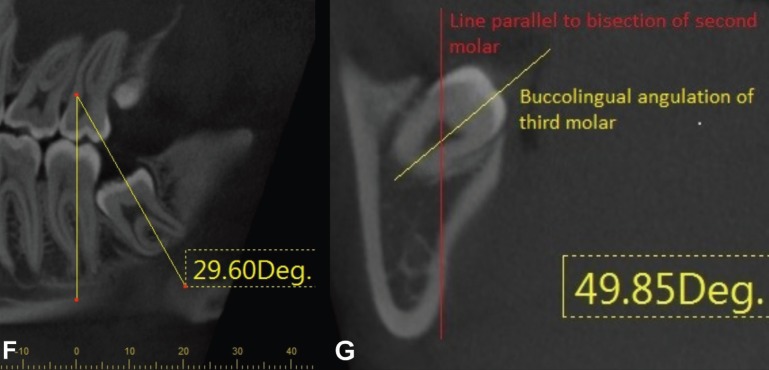
F-Mesio-distal angulation of the impacted third molar. G- Bucco-lingual angulation of the impacted third molar on an coronal CBCT slice.

The mesiodistal and buccolingual angulations of impacted third molars correlated with the thickness of the lingual cortical bone. Buccal, mesial angulations were given positive values and lingual, distal angulations have negative values. The mesiodistal angle of the impacted third molar was measured relative to the vertical axis of the erupted second molar (Fig. 3F). The buccolingual angulation was measured with a method previously described by Tolstunov et al (14). (Fig. 3G) Axial CBCT slices were used to measure the buccolingual angulation of third molars in a more horizontal position (-20◦ to 45◦, or 136◦ to 180◦), and coronal CBCT slices to measure teeth more vertically angulated (46◦ to 135◦) relative to the bisecting line through the second molar. Buccal incliation of the crown was given a plus value and lingual angulation a negative value. Buccolingual angulation was measured buccally or lingually from the axis bisecting the first and second molars in an axial CBCT slice for third molars in a more horizontal position. Coronal CBCT slices were used for more vertically angulated teeth and buccolingual angulation was measured respect to the bisecting line through the second molar (from the fossa through the middle of the pulp chamber). A line parallel to the second molar’s bisecting line was drawn and the difference in the angulation of the crown was calculated (Fig. 3G).

-Surgical procedure

All operations were performed by the same surgeon. Before the surgery, patients used 10% povidone-iodine mouthwash for one minute. Inferior alveolar nerve block and buccal infiltration were performed with 2 ml of 4% articaine HCl and 1:200,000 epinephrine solution. A full thickness mucoperiosteal flap was elevated. A conventional rotary handpiece and tungsten-carbide burs were used under irrigation for removing the overlying bone. If needed, the tooth was sectioned. Extraction wound was closed with 3-0 silk sutures. Postoperative antibiotic (1000 mg amoxycillin-clavulanic acid, bi-daily), analgesic therapy (flurbiprofen 100 mg, bi-daily) and antiseptic mouthwash (chlorhexidine gluconate,three times a day) were given. One week after surgery the sutures were removed and lingual nerve assessed. On the postoperative seventh day, the patients were asked following questions:

• Do you have a taste disorder?

• Do you have difficulty when speaking?

• Do you have numbness on your tongue?

-Statistical analysis

Statistical analysis was performed by SPSS version 21.0 (Statistical Package for Social Science Inc., Chicago, IL). Data were tested for normality using the Shapiro-Wilk test. Normality was violated and data were analyzed by the Wilcoxon, Mann-Whitney U test for gender differences and the Kruskal-Wallis H test for Winter angulations. The Pearson chi-square test was used for categorical variables. Descriptive statistics including mean and standard deviations were calculated for all variables. Spearman’s rho correlation coefficient test was used to investigate the correlation of mesiodistal and buccolingual angulation with the thickness of bone. *P* values less than 0.05 was considered to be significant.

## Results

The study consisted of 104 mandibular third molars of 104 patients. The mean age of the sample was 24.67±6.11 years. After surgeries, none of the 104 patients experienced paresthesia including the teeth with close proximity with lingual nerve. None of the patients reported taste disorders, speech difficulty or numbness on tongue. The mean thickness of bone at the CEJ of the second molar was 1.21±0.63 mm, at the mid-root of the third molar was 1.25±1.02 mm and at the apex of the third molar was 1.06±1.31 mm. The bone thickness was differed at only mid-root level between horizontal, mesioangular, distoangular and vertical angulations (p=0.007). The bone around distoangular and horizontal impactions was thinner than vertical and mesioangular third molars at mid-root level ( Table 1).

**Table 1:**
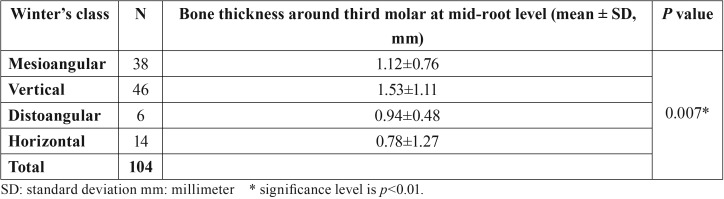
The mean bone thickness around third molars according to Winter classes of impacted third molars.

The thickness of lingual bone was found to be higher in females than males at mid-root and apex level (*p*<0.05) ( Table 2).

**Table 2:**
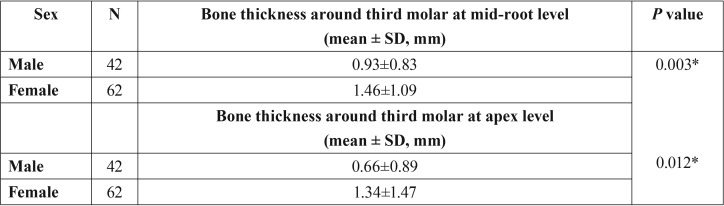
The mean bone thickness around third molars according to sex.

The mean buccolingual angulation was -5.91±14.35° and mesiodistal angulation was 26.85±35.34°. Thinning of the lingual bone (thickness<1mm) at mid-root and apex level was significantly associated with the buccolingual and mesiodistal angulation of third molar (*p*<0.05).

Buccolingual angulation of impacted teeth was significantly associated with lingual bone perforation (*p*=0.002, *p*<0.05). The mean buccolingual angulation of third molars associated with perforated bone was -0.49±13.07° compared with -10.22±13.95° when the bone was not perforated. The mean mesiodistal angulation of third molars associated with perforated bone was 33.88±36.66° compared with 21.29±33.53° when the bone was not perforated.

Spearman’s correlation coefficient showed that buccolingual and mesiodistal angulations of impacted tooth were negatively correlated with lingual bone thickness at mid-root and apex level (*p*<0.05). It was showed that when buccolingual and mesiodistal angulations increased, thickness of lingual bone decreased.

There was statistically significant relationship between Winter class of impacted third molar and lingual bone thinning at only mid-root level (*p*=0.016, *p*<0.05). Horizontally impacted teeth showed more lingual bone thinning at mid-root level than other impaction positions ( Table 3).

**Table 3:**
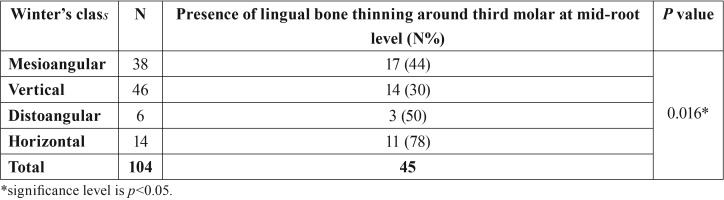
Distribution of lingual bone thinning according to the Winter’s class of the impacted third molar.

## Discussion

Lingual nerve damage is a possible complication of impacted lower third molar surgery (15). The lingual bone also helps protecting the lingual nerve. If the lingual bone is thin or fenestrated, risk of lingual nerve damage increases (14,16).

Angulation of the impacted tooth and thickness of the lingual bone may affect the difficulty of the surgery (17). Mallick *et al.* (18) evaluated thickness of lingual bone and lingual positions of lower third molars by using CBCT of 251 patients in a retrospective study. They reported that 4.4% of the cases were in contact with lingual cortical bone. In a retrospective study, a significant correlation was found between angulation of the tooth and lingual bone perforation. Thickness of lingual bone in middle third of horizontal and mesioangular impacted teeth was 3.6 times thinner than distoangular and vertical impacted teeth. In buccally angulated teeth, perforation was more common on middle and apical portions of the roots (14). In our study, we found that there was a significant correlation between buccolingual angulation and lingual bone perforation (*p*=0.002). This suggests that as the buccolingual angulation increases, the roots deviate towards the lingual side, so the lingual bone becomes thinner or perforated. According to Winter’s classification, lingual bone thickness was most affected at the middle portions of the roots (*p*=0.016). The cases in which the lingual bone was thinnest, were horizontal (78%) and distoangular (50%) teeth. As the mesiodistal angulation increased, the lingual bone became thinner.

We found that the lingual bone was significantly thicker in women at middle (*p*=0.003) and apical (*p*=0.012) portions of the roots. We thought, because the teeth of male patients tend to be larger, the lingual bone might become thinner. Thus, we may suggest that extra caution should be given to mesioangular and horizontal impacted lower third molars in male patients.

Based on the CBCT data, we could precisely identify the relationship between the root and the lingual bone. None of the 104 patients experienced taste disorders, speech problems or lingual nerve paresthesia. We also did not encounter displacement of the teeth or the roots through the lingual side to anatomical spaces.

CBCT cannot demonstrate soft tissues, including lingual nerve. This is the most important limitation of this study. We could only measure the thickness of the lingual bone.

## Conclusions

During impacted lower third molar surgery, lingual bone constitutes a natural barrier for the lingual nerve. According to our results, horizontal, distoangular and buccally angulated teeth are closer to the lingual nerve and that means a thinner lingual bone. To our knowledge, this is the first study investigating the relationship between thickness of the lingual bone and angulation of the impacted lower third molars and also lingual nerve paresthesia.
